# Harnessing plant metabolic pathways for innovative diabetes management: unlocking the therapeutic potential of medicinal plants

**DOI:** 10.1080/15592324.2025.2486076

**Published:** 2025-04-07

**Authors:** Ugwu Okechukwu Paul-Chima, Anyanwu Chinyere Nkemjika, Ugwu Melvin Nnaemeka, Hope Onohuean

**Affiliations:** a Department of Publication and Extension, Kampala International University, Ishaka-Bushenyi, Uganda; b Department of Microbiology and Immunology, Kampala International University, Ishaka-Bushenyi, Uganda; c Department of Medical Biochemistry, Faculty of Basic Medical Sciences, State University of Medical and Applied Science, Enugu, Nigeria; d Biomolecules, Metagenomics, Endocrine and Tropical Disease Research Group (BMETDREG), Kampala International University, Ishaka-Bushenyi, Uganda; e Biopharmaceutics unit, Department of Pharmacology and Toxicology, School of Pharmacy, Kampala International University, Ishaka-Bushenyi, Uganda

**Keywords:** Plant signaling pathways, diabetes management, medicinal plants, insulin resistance, oxidative stress, and gut microbiota modulation

## Abstract

The exploration of plant signaling pathways is transforming the way diabetes is managed, providing new, multi-target strategies for controlling this complex metabolic disorder. Medicinal plants are rich in bioactive compounds like phytohormones, flavonoids and polyphenols, which regulate key pathways including oxidative stress, inflammation, insulin resistance, and gut microbiota modulation. Research is emerging on the therapeutic potential of Momordica charantia, Cinnamomum verum and Trigonella foenum-graecum, which enhance insulin secretion, sensitivity and glucose homeostasis. These plant derived compounds, resveratrol and plant based insulin mimetics, not only address metabolic dysfunction but also offer holistic treatment for long term complications such as neuropathy and retinopathy. The development of precision medicine advances the tailoring of plant based therapies to individual metabolic responses, increasing efficacy and decreasing reliance on synthetic drugs with adverse side effects. Despite challenges of standardization, regulatory barriers, and limited clinical trials, incorporating medicinal plants into national diabetes management guidelines represents a cost effective and accessible option, particularly in resource limited settings. In this review, we highlight the importance of collaborative work across disciplines and the use of technologies such as artificial intelligence to speed research and optimize patient specific applications. The therapeutic power of plant signaling pathways is harnessed to develop sustainable, inclusive, and effective diabetes management strategies.

Plant signaling networks are extremely complex and offer great promise for revolutionizing the way diabetes is managed.^1^ Researchers are looking for new pathways that regulate blood glucose levels, by discovering novel plant-based signaling molecules, which could change current therapeutic strategies. Phytohormones and secondary metabolites in plants, for instance, are implicated in the control of insulin secretion and sensitivity and may be good therapeutic targets.^2^ Furthermore, recent studies have emphasized how medicinal plants have the ability to modulate critical pathways like insulin resistance, inflammation and oxidative stress can offer a promising strategies ion the control of diabetes.^3^

Plant-based signaling molecules may be harnessed to treat diseases more safely and efficiently, by targeting fundamental mechanisms like oxidative stress and metabolic dysfunction.^1^ Medicinal plants are rich source of flavonoids and polyphenols, which have antioxidant properties and may alleviate oxidative damage and improve cellular function in diabetic patients.^4^ Research into the signaling properties of other plants such as *Momordica charantia* (bitter melon) has also shown natural compounds that stimulate insulin secretion and help improve pancreatic function as shown in Figure 1.^5^ As a result of that, it offers a new approach to improve insulin production in diabetics.^6^

**Figure 1.**
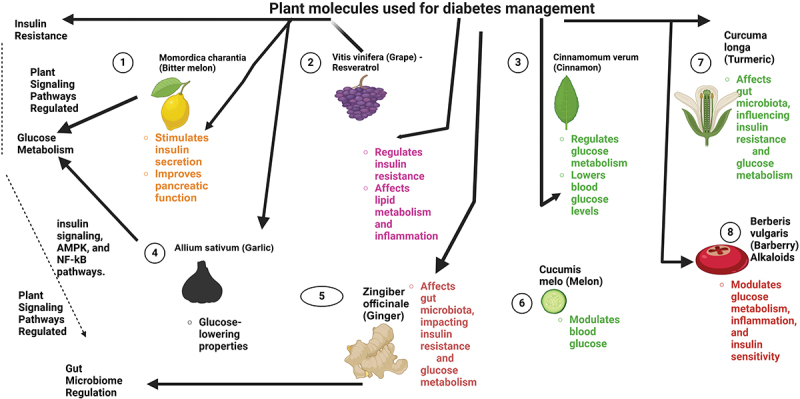
Plant molecules used for diabetes management.

Going deeper into plant signaling could also provide new biomarkers for diabetes and more tailored treatment strategies.^7^ Customized therapies for better disease management would, therefore, be possible if one understands the individual’s specific signaling responses.^8^ The incorporation of plant-based therapies in diabetes care can alleviate some degree of reliance on synthetic drugs and decrease side effects allowing for better outcomes for patients.^9^ Medicinal plants are known to control blood sugar naturally with fewer side effects than conventional diabetes medications such as metformin or sulfonylureas.^10^

Moreover, medicinal plants are able to regulate complex signaling pathways addressing both the symptoms and the root cause of diabetes.^11^ Resveratrol in *Vitis vinifera* (grape) is one of the plant derived compounds that regulate insulin resistance, lipid metabolism and inflammation.^12^ Targeting of these pathways can improve insulin sensitivity and, hopefully, provide more holistic management strategies for type 2 diabetes.^13^ In addition, plant-based therapies can treat long-term diabetes complications including neuropathy and retinopathy.^14^ Compounds found in *Camellia sinensis* (green tea) containing polyphenols possess promising anti-inflammatory and microvascular health improvement qualities which may assist in the management of diabetic complications.

Furthermore, in addition, plant signaling pathways are also an extension of the therapeutic power of the plants.^15^ Research shows that plant-based insulin mimetics, such as those found in *Trigonella foenum-graecum* (fenugreek), may be a natural alternative to insulin for patients with insulin-dependent diabetes.^16^ Without side effects, this could allow accessible treatments especially in areas with little or no access to conventional medications.^17^ Traditional medicinal plants are used by many regions to manage diabetes and *Cinnamomum verum* (cinnamon) and *Allium sativum* (garlic) are well known for their glucose lowering properties as shown in Figure 1.^18^ Not only do these plants regulate metabolic pathways, but they provide a cost effective, natural solution to the global diabetes epidemic.^19^

In addition, research into plant signaling is revealing how precision medicine could be used to treat diabetes as shown in the graphical abstract.^20^ Plant compounds such as those in *Allium cepa* (onion) work by regulating glucose metabolism and insulin sensitivity via specific targeting metabolic pathways and can, therefore, be used as means to create more targeted and effective treatments.^21^ Likewise, plant blood glucose modulators such as *Cucumis melo* and *Pterocarpus marsupium* may work to round out a potential affordable and accessible treatment in low-income regions.^22^ A deeper understanding of how medicinal plants affect key signaling molecules that control blood glucose homeostasis may hold the key to diabetes management in the future.^23^

**Figure:**
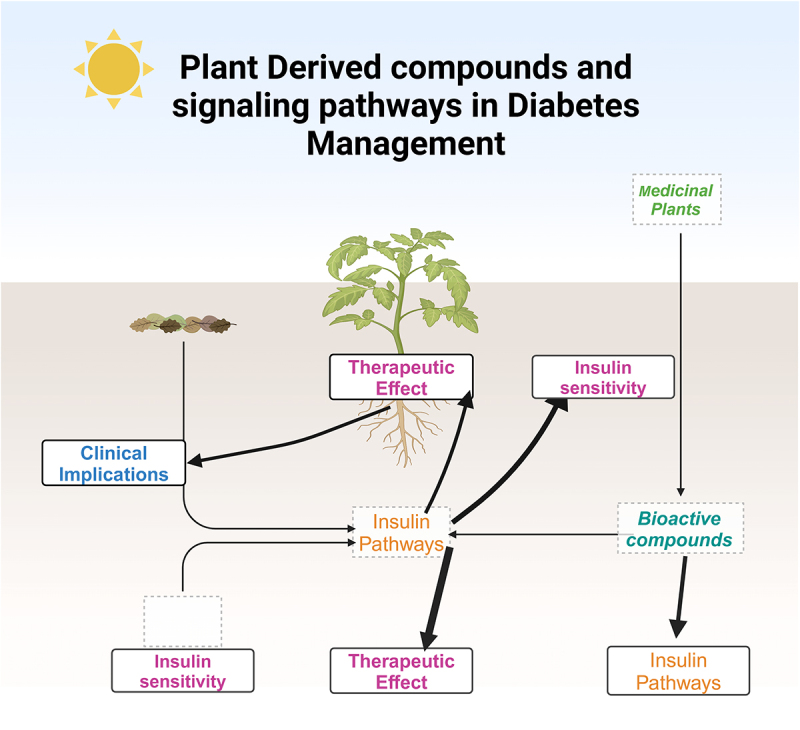

Regulation of gut microbiome, an emerging target in diabetes management, also holds promise in plant signaling.^19^ Some medicinal plants such as *Zingiber officinale* (ginger) and *Curcuma longa* (turmeric) affect gut microbiota, which, in turn, affects insulin resistance and glucose metabolism.^24^ Modulation of these pathways not only allow plants to regulate glucose levels but also to promote overall metabolic health and thus confer broader benefits to diabetic patients.^25^ Moreover, further studies on how medicinal plants control the absorption of glucose and insulin sensitivity could help in new dietary strategies in preventing type 2 diabetes.^25^

Plant composition is highly variable and is affected by cultivation conditions and processing methods, making standardization and reproducibility in therapeutic applications extremely challenging.^26^ Other aspects of the complex drug interactions need to be examined as well, especially identifying potential side effects like interactions with regular medicines or problems of toxicity that might come from high doses.^27^ Obstacles to integrating plant-based therapies include regulatory barriers, limited clinical trials and skepticism from healthcare providers.^28^ These factors illuminate the importance of working heavily with rigorous research as well as collaboration between traditional and modern medical systems to fill in that gap.^26^ In addition, using advanced technologies like artificial intelligence (AI) can help in understanding of the patient-specific responses to plant-based compounds and personalize the treatments.^27^ It is recommended that plant-based therapies can be integrated into national diabetes management guidelines and that initiatives that fund research in medicinal plants be supported.^28^ These types of measures could help create a more inclusive approach to diabetes care that ensures its accessibility and sustainability in a variety of contexts of care.^29^

Studies show that plant-based therapies have similar or better prospective than conventional treatments in type 2 diabetes. For instance, the fasting blood glucose is reduced by around 30–40% after intake of *Momordica charantia* (bitter melon), as compared to 10–20% with metformin.^5^ Moreover, less gastrointestinal side effects are reported with bitter melon extract which are accompanied with metformin uptake, suggesting it as a more sustainable option in long-term use.^30^ *Cinnamomum verum* (cinnamon) has also been shown to enhance insulin sensitivity. A meta-analysis of 10 RCTs found HbA1c reductions of 0.5–1.0% which is similar to that seen with oral hypoglycemic agents such as sulfonylureas or thiazolidinediones but without the side effects seen with those medications such as weight gain or hypoglycemia.^31^ *Trigonella foenum-graecum* (fenugreek) lowers postprandial blood glucose by approximately 25–30% in clinical studies,^32^ which is similar to what can be achieved with many of the newer diabetes medications such as DPP-4 inhibitors or SGLT2 inhibitors that have come to market by their ability to reduce hyperglycemia.^32^ Resveratrol, a compound found in *Vitis vinifera* (grape), was found to reduce insulin resistance by 20–30 is comparable with the action of pioglitazone but without the weight gain and edema associated with pioglitazone.^33^ This preliminary finding points toward the potential role for plant-based compounds as adjuvants or even alternatives to existing therapeutics, particularly for patients intolerant to conventional pharmacological agents mainly due to side effects.^34^ However, metformin and insulin will continue as cornerstones of therapy. Adjunctive plant-based compounds could be used as alternative treatments that are less costly and have fewer associated complications than conventional drugs for a range of conditions including diabetes.^17^ Adjuvant treatment would also provide additional benefits via their antioxidant and anti-inflammatory properties leading potentially to less complications such as neuropathy and retinopathy in diabetic patients.^19^ Nevertheless, there remain many challenges ahead such as determining appropriate doses, lack of availability of key active ingredients in some regions/countries/international suppliers/are issues that need further elucidation before implementation can occur on a wider scale with this type of therapy.^19^ An optimal integrative model utilizing both biomedical advances alongside classical tradition practices for diabetes management has been proposed with each approach augmenting the deficiencies inherent in one another as suggested.^35^

By integrating plant signaling pathways into diabetes management, novel strategies controlling multiple disease pathways at a time could be unlocked.^36^ *Berberis vulgaris* (barberry) is a plant which contains the alkaloids that modulate the key pathways involved in glucose metabolism, inflammation and insulin sensitivity.^37^ This opens up new possibilities for developing multi-target therapies that address the complex pathophysiology of the disease.^38^ Finally, plant signaling knowledge provides an exciting new frontier for natural effective treatments developments in diabetes care.^1^ Plant-based therapies could transform the way diabetes is treated by restoring balance to the body’s metabolic networks, making it more effective, more accessible and more sustainable.^39^

The promising therapeutic value of plant-based signaling molecules in diabetes management requires additional research to achieve full clinical application.^40^ Metabolic engineering provides a crucial solution to increase yield and consistency levels of bioactive compounds extracted from plants.^41^ The field requires technological advancements to achieve better control of plant-based product diversity because current challenges limit easy manufacturing of standard plant-based compounds.^41^ The development of these efforts will generate reliable plant-derived compounds of increased potency which are better suited for therapeutic applications.^42^ Transgenic technologies provide potential solutions to enhance plant molecule synthesis and effectiveness through genetic modification which produces higher quantities of specific compounds.^43^ Genetically modified plants created through this method would consistently generate elevated levels of bioactive substances, thereby making them available for widespread large-scale production which supports diabetes management.^43^ The clinical validation of plant-derived drugs for diabetes treatment needs stronger evidence based on preclinical results.^41^ Clinical research needs to conduct extensive trials that determine safe therapeutic amounts and examine both short-term and long-term effects of plant-based medical treatments.^42^ The integration of plant-derived compounds into conventional diabetes care requires thorough examination of drug interactions together with potential side effects to establish their safe utilization.^47^ Individualized medical approaches based on metabolic profiling reveal additional advantages regarding the best utilization of *Trigonella foenum-graecum* (fenugreek) and *Cinnamomum verum* (cinnamon) compounds for therapeutic benefits.^44^ Research of altered signaling pathways in diverse diabetic subtypes will help develop exact and customized therapeutic approaches which improve medical results specifically for each patient type.^45^ Patient-specific data examination through AI techniques enables efficient creation of personalized treatment protocols that match diabetic patient-specific metabolic characteristics.^46^ Plant-based therapies for diabetes management require decisive solutions to improve metabolic engineering and transgenics along with better clinical trial results to achieve forward progress.^47^ The combination of traditional herbals knowledge with contemporary scientific methodology enables more practical cost-effective and accessible medical treatments primarily for healthcare-deserted areas.

## Data Availability

Additional data shall be made available by the author on request.
